# National Trends and Outcomes of Combined Lung–Liver Transplantation: An Analysis of the UNOS Registry

**DOI:** 10.1007/s00408-025-00811-9

**Published:** 2025-04-25

**Authors:** Brian J. Bao, Ye In Christopher Kwon, Emily G. Dunbar, Zachary Rollins, Jay Patel, Matthew Ambrosio, David A. Bruno, Vipul Patel, Walker A. Julliard, Vigneshwar Kasirajan, Zubair A. Hashmi

**Affiliations:** 1 Division of Cardiothoracic Surgery, Department of Surgery, Pauley Heart Center, Virginia Commonwealth University School of Medicine, Richmond, VA USA; 2 Department of Biostatistics, Virginia Commonwealth University School of Population Health, Richmond, VA USA; 3 Division of Abdominal Transplant Surgery, Department of Surgery, Hume-Lee Transplant Center, Virginia Commonwealth University School of Medicine, Richmond, VA USA; 4 Division of Pulmonary Disease and Critical Care Medicine, Department of Internal Medicine, Virginia Commonwealth University School of Medicine, Richmond, VA USA

**Keywords:** Combined lung–liver transplant, Lung transplant, Liver transplant, Lung allocation score, UNOS registry, Idiopathic pulmonary fibrosis

## Abstract

**Purpose:**

Combined lung–liver transplant (CLLT) is a complex yet life-saving procedure for patients with simultaneous end-stage lung and liver disease. Given the geographical allocation change to the lung allocation score (LAS) in 2017 and the recent SARS-CoV-2 outbreak in 2019, we aim to provide an updated analysis of the patient selection and outcomes of CLLTs.

**Methods:**

The UNOS registry was used to identify all patients who underwent CLLT between January 2014 and June 2023. To account for the changes made to LAS in 2017, baseline characteristics and outcomes were compared between era 1 (before 2017) and era 2 (after 2017). Risk factors for mortality were analyzed using the Cox regression hazard models. Recipient survival of up to 3 years was analyzed using the Kaplan–Meier method.

**Results:**

117 CLLTs were performed (77.8% in era 2). Donor organs experienced significantly longer ischemic times (*p* = 0.039) and traveled longer distances (*p* = 0.025) in era 2. However, recipient (*p* = 0.79) and graft (*p* = 0.41) survival remained comparable at up to 3 years post-transplant between eras. CLLTs demonstrated similar long-term survival to isolated lung transplants (*p* = 0.73). Higher recipient LAS was associated with an increased mortality risk (HR 1.14, *p* = 0.034)*.* Recipient diagnosis of idiopathic pulmonary fibrosis carried a 5.03-fold risk of mortality (*p* = 0.048) compared to those with cystic fibrosis.

**Conclusion:**

In the post-2017 LAS change era, CLLTs are increasingly performed with comparable outcomes to isolated lung transplants. A careful, multidisciplinary approach to patient selection and management remains paramount to optimizing outcomes for this rare patient population.

**Supplementary Information:**

The online version contains supplementary material available at 10.1007/s00408-025-00811-9.

## Introduction

Despite its complexity and rarity, combined lung–liver transplantation (CLLT) can be a life-saving procedure in patients with concomitant end-stage lung and liver disease, especially those who are not predicted to survive isolated lung or liver transplantation [1–3]. While the pathophysiology of liver dysfunction in lung transplant patients remains unclear, extensive use of cardiopulmonary bypass and the associated catecholamine release may be related to the development of post-transplant hypoxic hepatitis [4]. The most common indication for CLLT is cystic fibrosis (CF) with concomitant liver cirrhosis. As such, much of the early CLLTs were performed in pediatric patients [5]. However, the demand for CLLTs among adults has steadily risen, particularly among those with alpha-1-antitrypsin deficiency (A1 AD), idiopathic pulmonary fibrosis (IPF), and sarcoidosis [6, 7].

Outcomes of CLLTs have seen tremendous improvements. As of the 2010s, reports have indicated 1-year survival rates as high as 92% [5, 6, 8–11]. These results are likely due to patient selection and risk stratification improvements primarily driven by the lung allocation score (LAS) [6]. However, due to the limited number of cases and center variability in patient selection or perioperative management, long-term outcomes of CLLTs have seldom been analyzed nationally. Thus, there is an ongoing debate about whether the long-term benefits of CLLT warrant the simultaneous allocation of both organs to a single recipient [12]. The most recent national analysis of CLLT outcomes ended in 2016 and demonstrated no differences in survival between CLLT and isolated lung transplants at up to 5-year post-transplant [3, 13]. However, in the context of the SARS-CoV-2 outbreak in 2019 (COVID-19) [14] and the geographic unit changes to the LAS in 2017 [15, 16], the outcomes of CLLTs have yet to be determined. Thus, we aimed to provide an updated analysis of recipient and donor selection, clinical outcomes, and associated risk factors in CLLTs.

## Materials and Methods

### Source of Data

This study utilized the United Network for Organ Sharing (UNOS) Standard Analysis and Research (STAR) database. Because all data were de-identified, it was deemed exempt from the Virginia Commonwealth University Institutional Review Board. It also complies with the International Society for Heart and Lung Transplantation (ISHLT) ethics policies.

### Study Population

The OPTN/UNOS STAR file was reviewed to identify records of all adult patients (aged ≥ 18 years) listed for first-time lung transplantation in the USA from January 1, 2014 to June 30, 2023. Then, patients who received lungs and livers from the same donors were included. On November 24, 2017, the UNOS/OPTN changed the LAS to distribute lungs first within a 250-nautical mile radius from the donor hospital [15]. To better understand the impact of this change on patient selection and outcomes, the overall cohort was divided into two eras: January 1, 2014 to November 24, 2017 (era 1) and November 25, 2017 to June 30, 2023 (era 2).

### Statistical Analysis

Comprehensive donor and recipient demographics, waitlist times, comorbidities, and preoperative medical and hemodynamic characteristics were collected, with categorical variables reported as percentages and continuous variables reported as medians and interquartile ranges (IQR). Patient survival at 30 days, 1, and 3 years was the primary outcome of interest and was defined as the length of time from the date of CLLT until the date of last patient contact or death. Secondary outcomes were primary graft survival at 30 days, 1, and 3 years after CLLT, length of hospital stay, rates of postoperative infection requiring hospitalization, acute rejection, airway dehiscence, postoperative dialysis, stroke, and reintubation. Differences between eras were determined by Chi-square or Fisher’s exact tests for categorical variables and the Kruskal–Wallis test for continuous variables. The Kaplan–Meier method was used to plot and compare the overall survival of all adult CLLT and isolated lung transplant recipients. Adjusted multivariable cox-proportional hazard regression was performed to assess significant predictors of overall survival. Covariates included recipient age, sex, diabetes, Model for End-Stage Liver Disease (MELD-XI) scores, LAS, smoking status, waitlist time, indication, donor age, and donor left ventricular ejection fraction. 95% confidence intervals (CI) were reported for all outcomes. All statistical analyses were conducted using R (version 4.3.0). All *p *values were based on two-sided statistical tests, with significance set at *p* < 0.05.

## Results

### Baseline Donor and Recipient Details

**Fig. 1:**
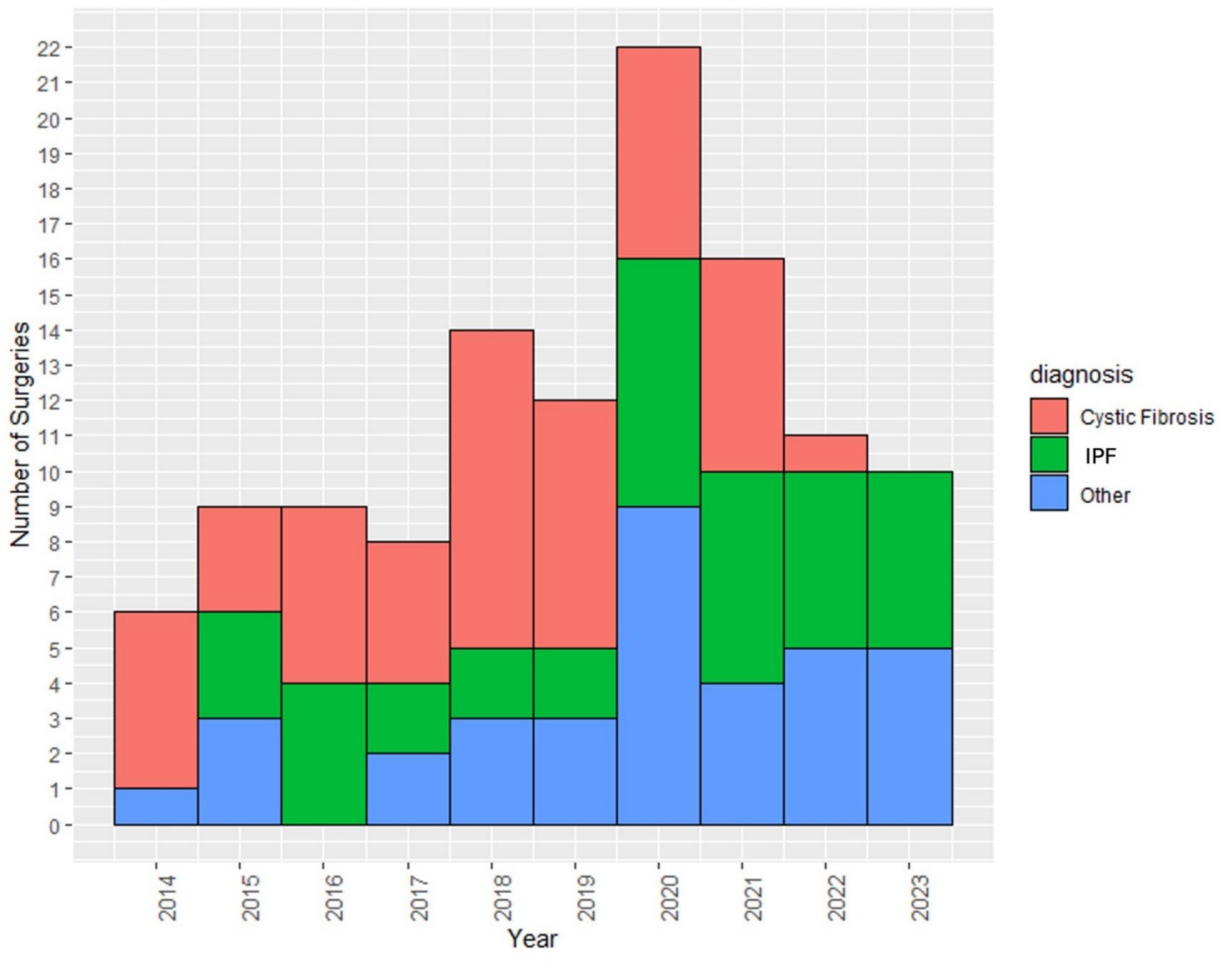
Trends in the number of combined lung–liver transplantations performed in the USA by year, separated by indication for surgery. As data collection ended in June 2023, data from 2023 are incomplete. IPF stands for interstitial pulmonary fibrosis

Since 2014, the number of CLLTs being performed has steadily risen, with a noticeable peak in 2020 (Fig. 1). We identified 117 patients who underwent CLLT during the study period. A total of 84 (71.8%) patients were transplanted during era 2 (Table 1). Notably, CLLT recipients in era 2 had higher body mass index (BMI) than those in era 1 (*p* = 0.01). In era 2, CLLT recipients had higher forced expiratory volume in one second (FEV1) (*p* = 0.022) and had higher pretransplant creatinine (*p* = 0.007) compared to those in era 1. We observed no significant differences in recipient age (*p* = 0.072), sex (*p* = 0.3), race (*p* = 0.086), and LAS (*p* = 0.7) between eras. Although statistically insignificant, CLLT recipients in era 2 were more likely to have higher LAS scores between 36 and 46 (48% vs. 54%) and 47–60 (9.1% vs. 14%). There were no significant differences in the recipients’ MELD-XI scores between eras (*p* = 0.7). Cystic fibrosis remains the predominant indication in both eras, accounting for 52% and 35% of CLLTs in eras 1 and 2, respectively. However, in era 2, more recipients required CLLTs due to IPF (27% vs. 32%), obstructive lung disease (0% vs. 4.8%), sarcoidosis (0% vs. 7.1%), and pulmonary hypertension (0% vs. 7.1%). Two patients required CLLT due to COVID-19-related acute respiratory distress syndrome (ARDS) or pulmonary fibrosis.

**Table 1:** Baseline recipient and donor demographics and characteristics of all adult recipients of combined lung liver transplantation by era

Variables	Overall, *N* = 117^a^	Era 1, *N* = 33^a^	Era 2, *N* = 84^a^	*p *value^b^
Recipient characteristics				
Female sex, *n* (%)	35 (30)	12 (36)	23 (27)	0.3
Age (years), median (IQR)	51.00 (29.00, 61.00)	44.00 (26.00, 58.00)	52.00 (32.50, 61.25)	0.072
BMI (kg/m^2^), median (IQR)	23.40 (19.80, 27.50)	21.70 (18.70, 23.70)	24.90 (20.13, 28.30)	**0.01**
Race, *n* (%)				0.086
White	94 (80)	32 (97)	62 (74)	
Asian	3 (2.6)	0 (0)	3 (3.6)	
Black	3 (2.6)	0 (0)	3 (3.6)	
Hispanic	16 (14)	1 (3.0)	15 (18)	
Other	1 (0.9)	0 (0)	1 (1.2)	
Indication, *n* (%)				0.2
Cystic fibrosis	46 (39)	17 (52)	29 (35)	
Idiopathic pulmonary fibrosis	36 (31)	9 (27)	27 (32)	
Obstructive lung disease	4 (3.4)	0 (0)	4 (4.8)	
Alpha-1-antitrypsin deficiency	6 (5.1)	3 (9.1)	3 (7.1)	
Sarcoidosis	3 (2.6)	0 (0)	3 (7.1)	
Pulmonary hypertension	3 (2.6)	0 (0)	3 (7.1)	
Secondary pulmonary hypertension	1 (0.9)	0 (0)	1 (1.2)	
Porto-pulmonary hypertension	1 (0.9)	0 (0)	1 (1.2)	
COVID-19 ARDS	1 (0.9)	0 (0)	1 (1.2)	
COVID-19 pulmonary fibrosis	1 (0.9)	0 (0)	1 (1.2)	
Other	15 (12.8)	4 (12)	11 (14)	
Diabetes, *n* (%)	48 (41)	17 (52)	31 (37)	0.15
History of cigarette use, *n* (%)	40 (34)	11 (33)	29 (35)	> 0.9
Dialysis, *n* (%)	4 (3.4)	2 (6.1)	2 (2.4)	0.3
On ventilator at transplant, *n* (%)	9 (7.7)	4 (12)	5 (6.0)	0.3
FEV1 (% of predicted), median (IQR)	36.50 (24.00, 54.00)	28.00 (21.00, 49.00)	42.00 (25.00, 58.00)	**0.022**
MELD-XI scores, median (IQR)	11 (6, 12)	10 (8,11)	11 (7,12)	0.7
Serum creatinine, median (IQR)	0.71 (0.61, 0.92)	0.62 (0.57, 0.80)	0.80 (0.64, 1.00)	**0.007**
Bilirubin, median (IQR)	1.00 (0.50, 1.90)	1.10 (0.50, 2.30)	0.95 (0.50, 1.78)	0.4
Lung allocation score, median (IQR)	38.15 (35.59, 47.28)	37.61 (35.27, 47.93)	38.43 (35.82, 47.06)	0.7
Lung allocation score categories, *n* (%)				0.6
< 24	2 (1.8)	1 (3.0)	1 (1.3)	
24–30	1 (0.9)	1 (3.0)	0 (0)	
30–35	20 (18)	6 (18)	14 (18)	
36–46	59 (52)	16 (48)	43 (54)	
47–60	14 (12)	3 (9.1)	11 (14)	
61–100	17 (15)	6 (18)	11 (14)	
Time on waitlist (days), median (IQR)	69.00 (25.00, 260.00)	69.00 (28.00, 245.00)	71.00 (24.75, 287.75)	> 0.9
Donor characteristics				
Female sex, *n* (%)	37 (32)	11 (33)	26 (31)	0.8
Age (years), median (IQR)	27.00 (20.00, 36.00)	26.00 (19.00, 37.00)	28.00 (20.00, 36.00)	0.3
BMI (kg/m^2^), median (IQR)	24.69 (21.50, 26.73)	23.88 (20.72, 25.25)	25.06 (22.03, 27.39)	0.2
Race, *n* (%)				0.07
White	74 (63)	27 (82)	47 (56)	
Asian	7 (6.0)	1 (3.0)	6 (7.1)	
Black	26 (22)	3 (9.1)	23 (27)	
Hispanic	10 (8.5)	2 (6.1)	8 (9.5)	
Left ventricular ejection fraction, median (IQR)	60.00 (55.00, 65.00)	55.00 (49.00, 65.00)	60.00 (55.00, 65.00)	0.12
Diabetes, *n* (%)	4 (3.4)	0 (0)	4 (4.8)	0.6
Ischemic time (hours), *n* (%)	5.00 (4.08, 6.05)	4.60 (3.80, 5.60)	5.30 (4.30, 6.30)	**0.039**
Donor-recipient distance (nautical miles), median (IQR)	103.00 (19.00, 212.00)	50.00 (10.00, 198.00)	112.00 (44.25, 227.00)	**0.025**

Bolded p-values indicate statistical significance at *p* < 0.05

*ARDS* acute respiratory distress syndrome, *BMI* body mass index, *FEV1* forced expiratory volume in one second, *MELD-XI* model for end-stage liver disease

^a^n (%); median (IQR)

^b^Pearson’s Chi-squared test; Fisher’s exact test; and Wilcoxon rank sum test

Donors of both the lungs and the liver were similarly matched between eras, with no significant differences in sex (*p* = 0.8), age (*p* = 0.3), BMI (*p* = 0.2), and race (*p* = 0.07). However, CLLT donor organs in era 2 experienced longer ischemic times (5.3 vs. 4.6 h, *p* = 0.039) than those in era 1. Similarly, CLLT donor organs in era 2 traveled further to reach their matched recipients (112 vs. 50 nautical miles, *p* = 0.025) compared to those in era 1.

### Clinical Outcomes

**Fig. 2:**
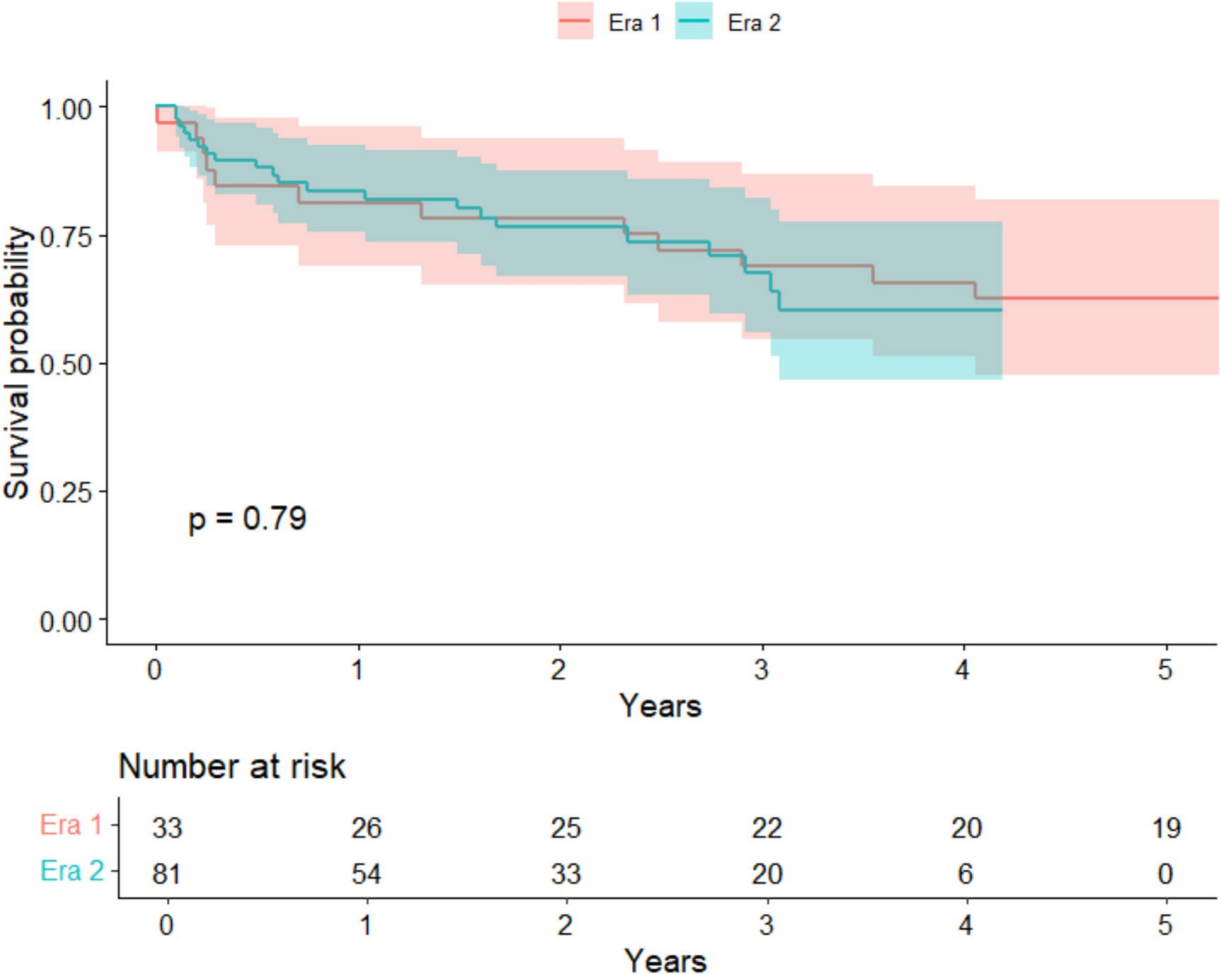
Kaplan–Meier survival curve for all adults undergoing combined lung–liver transplantation in the USA. The shaded region represents the 95% confidence interval

**Fig. 3:**
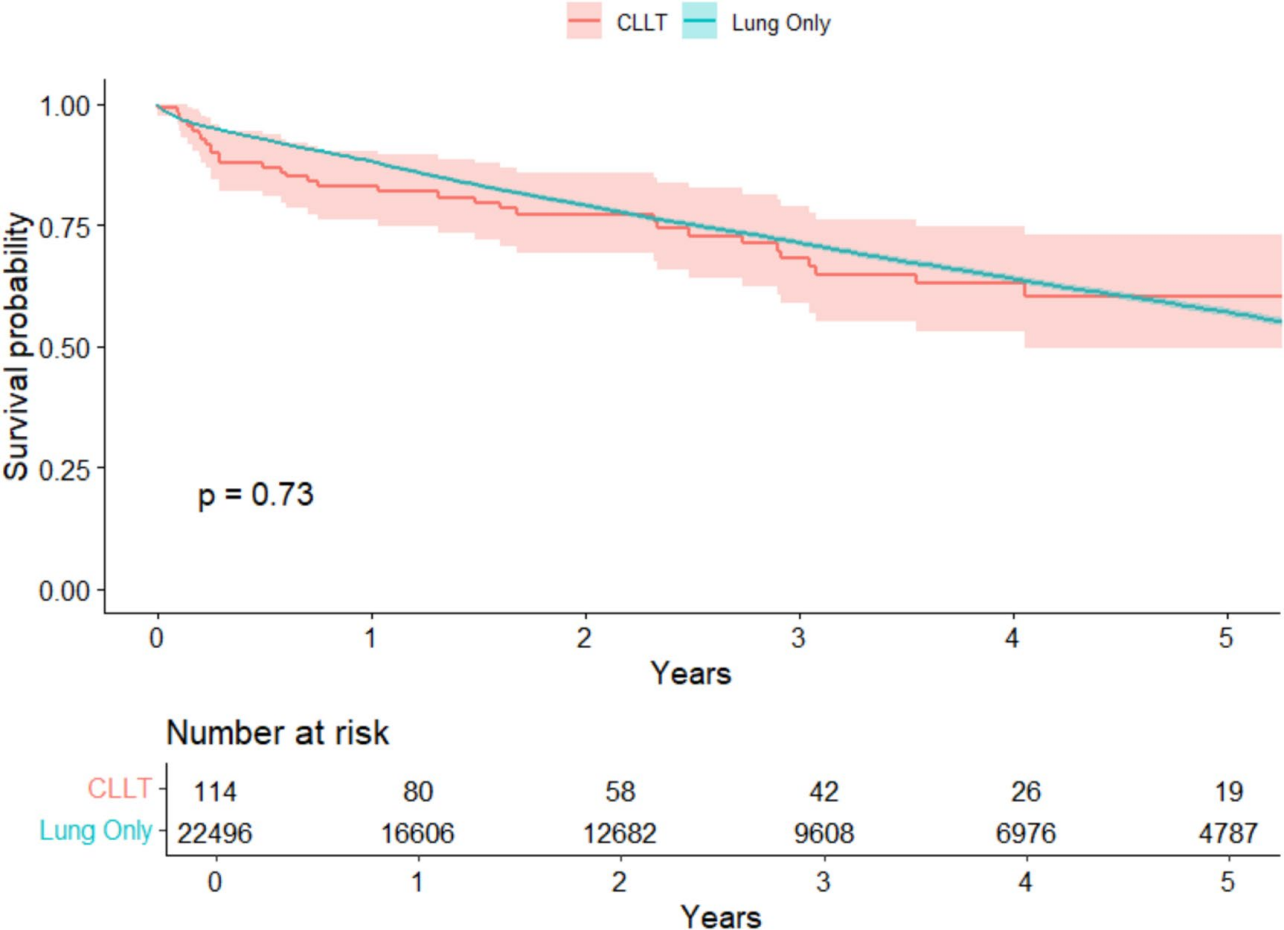
Kaplan–Meier survival curve comparing all adults undergoing combined lung–liver and isolated lung transplantation in the USA. The shaded regions represent the 95% confidence interval

We observed no significant differences in recipient survival between eras at 30-day, 1-, and 3-year intervals (*p* = 0.79, Fig. 2). Lung allograft survival remained comparable between eras at the same time intervals (*p* = 0.41, Table 2). Rates of acute rejection (*p* = 0.7), airway dehiscence (*p* = 0.5), dialysis (*p* > 0.9), stroke (*p* = 0.4), and reintubation (*p* > 0.9) did not differ significantly between eras. However, the rates of postoperative infection requiring hospitalization were significantly reduced in era 2 (30% vs. 52%, *p* = 0.027). Recipient survival up to 5-year post-transplant remained comparable between CLLT and isolated lung transplantation during the entire study period (*p* = 0.73, Fig. 3). Within era 1, CLLT demonstrated comparable 5-year survival to isolated lung transplants (*p* = 0.26, Fig. S1). Similarly, within era 2, CLLT showed similar 3-year survival compared to isolated lung transplant (*p* = 0.7, Fig. S2). Supplemental figures can be found in Online Resource 1.

**Table 2:** Clinical outcomes of all adult recipients of combined lung–liver transplant by era

Outcomes	Overall, *N* = 117^a^	Era 1, *N* = 33^a^	Era 2, *N* = 84^a^	*p*-value^b^
Recipient survival, % (94% CI)				0.79
30 days	99.1 (97.4, 100)	97.0 (91.3, 100)	100 (100, 100)	
1 year	82.9 (76, 90.5)	81.3 (68.9, 96.0)	83.6 (75.5, 92.6)	
3 years	68.2 (58.9, 78.9)	68.8 (54.5, 86.9)	67.7 (55.8, 82.1)	
Lung allograft survival, % (94% CI)				0.41
30 days	98.2 (95.8, 100)	93.9 (86.1, 100)	100 (100, 100)	
1 year	82.2 (75.2, 89.8)	78.8 (66.0, 94.0)	83.6 (75.5, 92.6)	
3 years	67.6 (58.3, 78.3)	66.7 (52.4, 84.9)	67.7 (55.8, 82.1)	
Acute rejection, *n* (%)	7 (6.2)	1 (3.0)	6 (7.5)	0.7
Postoperative infection requiring hospitalization, *n* (%)	42 (36)	17 (52)	25 (30)	**0.027**
Length of hospital stay, days, median (IQR)	29.00 (21.00, 54.00)	30.00 (22.75, 75.00)	28.50 (21.00, 52.00)	0.3
Airway dehiscence, *n* (%)	2 (1.7)	1 (3.0)	1 (1.2)	0.5
Dialysis, *n* (%)	21 (18)	6 (18)	15 (18)	> 0.9
Stroke, *n* (%)	7 (6.0)	3 (9.1)	4 (4.8)	0.4
Reintubation, *n* (%)	28 (24)	8 (24)	20 (24)	> 0.9

Bolded p-values indicate statistical significance at *p* < 0.05

^a^n (%); median (IQR)

^b^Pearson’s Chi-squared test; Fisher’s exact test; and Wilcoxon rank sum test

### Risk Factors

Risk modeling by Cox regression analyses revealed several notable independent predictors of mortality after CLLT (Table 3). Notably, the era of transplant had no impact on survival (HR 3.03, *p* = 0.1). Recipient diabetes was associated with a 2.68-fold increased risk of mortality (*p* = 0.021). Patients diagnosed with IPF were at a 5.03-fold increased risk of mortality following CLLT (*p* = 0.048) compared to those diagnosed with CF. Similarly, recipients diagnosed with all other indications for CLLT were at a 6.78-fold increased risk of mortality (*p* = 0.014). These indications included obstructive lung diseases (including emphysema and bronchiectasis), A1 AD, sarcoidosis, pulmonary hypertension, secondary pulmonary hypertension, porto-pulmonary hypertension, and COVID-19-related ARDS or pulmonary fibrosis.

**Table 3:** Multivariate cox regression analysis for predictors of recipient survival after combined lung–liver transplantation

Variable	Hazard ratio	95% Confidence interval	*p*-value
Era of transplant (era 1 as reference)	3.03	0.78–3.52	0.10
Recipient female sex (male as reference)	0.98	0.91–1.14	0.95
Recipient age	0.97	0.93–1.01	0.107
Recipient diabetes	2.68	1.16–6.15	**0.021**
Recipient history of cigarette use	1.08	0.87–1.25	0.85
Recipient MELD-XI score	1.05	0.99–1.12	0.122
Recipient LAS	1.14	1.10–1.24	**0.034**
Recipient LAS categories (< 24 as reference)			
24–30	0.74	0.81–1.20	0.27
30–35	1.05	0.95–1.78	0.78
36–46	1.27	0.86–2.01	0.65
47–60	1.67	1.05–2.26	**0.042**
61–100	2.14	1.25–3.75	**0.015**
Recipient days on waitlist	1.27	1.08–1.31	**0.02**
Recipient diagnosis (CF as reference)			
IPF	5.03	1.02–24.85	**0.048**
Other	6.78	1.48–31.05	**0.014**
Donor age	0.97	0.93–1.00	0.066
Donor ejection fraction	1.01	0.92–1.09	0.48

Bolded p-values indicate statistical significance at *p* < 0.05

*CF* cystic fibrosis, *IPF* idiopathic pulmonary fibrosis, *LAS* lung allocation score, *MELD-XI* model for end-stage liver disease

While recipient MELD-XI scores were not associated with mortality (HR 1.05, *p* = 0.122), recipients with higher LAS scores had a 1.14-fold increased mortality risk after CLLT (*p* = 0.034). Specifically, LAS score ranges of 47 to 60 (HR 1.67, *p* = 0.042) and 61 to 100 (HR 2.14, *p* = 0.015) were associated with an increased mortality risk after CLLT. Furthermore, longer waitlist time for recipients was associated with a 1.27-fold increased mortality risk (*p* = 0.02).

## Discussion

This study represents one of the largest and most updated retrospective analyses of the trends and long-term outcomes of CLLTs performed in the USA. We demonstrate that CLLTs have been increasingly performed with comparable long-term survival to isolated lung transplantation, indicating that the lung allografts may determine these patients’ long-term outcomes. Prior studies have demonstrated notable escalation in case numbers, particularly after 2006, with a significant spike in 2009 [12]. This initial increase is likely attributed to the implementation of the LAS in 2005, which shifted the primary criterion for lung allocation from waiting time to medical need. This shift roughly tripled the rate of CLLT among candidates [12]. A more recent spike in cases occurred in 2020, possibly because of the profound impact of the COVID-19 pandemic on lung function and, to a lesser extent, liver health. Yet only a few patients underwent CLLT solely due to COVID-19-related ARDS. Thus, the implications of the pandemic on CLLT outcomes in the post-COVID-19 era remain subject to ongoing evaluation.

In the past, several single-center case series have shown a steady improvement in long-term survival, surpassing the cumulative outcomes of our nationwide analysis spanning from 2014 to the present. 1-year survival rates in the 1990 s and 2000 s ranged from 56 to 79%, with 5-year survival rates in the same period ranging from 49 to 63% [5, 8–10, 17]. Since 2011, however, specific centers have reported 1- and 5-year survival rates as high as 100% and 80%, respectively [7, 11, 18]. These single-center findings demonstrate a promising trend of improved survival rates that is not necessarily reflected in the outcomes reported in our nationwide analysis. As single-center case series continue to outperform nationwide averages, collaborative efforts are warranted to identify and disseminate best practices that contribute to improved CLLT outcomes.

Our study is also the first to demonstrate that since the geographic allocation changes to the LAS in 2017, the recipient and graft survival after CLLTs have remained similar up to 3-year post-transplant. This is despite the increased ischemic times and distance between the donor and the recipient in the post-2017 era. These increases are expected, as the new primary lung allocation unit became a 250-nautical mile radius surrounding the donor hospital rather than the local donation service area [16]. As such, the ischemic times and the average distance between the transplant center and the donor hospital have significantly increased since the geographic allocation change for lung transplants [19]. However, despite similar post-transplant survival before and after this change, days on the waitlist have remained stagnant in our analysis. Wolf et al., albeit before the geographic allocation change, demonstrated significantly poorer survival for candidates on the CLLT waitlist compared to isolated lung waitlist candidates [20]. A more recent analysis has largely supported these results up to 2016 [3]. Importantly, they noted that the LAS for CLLT candidates and recipients was not significantly different from those of isolated lung transplants [3]. Our study demonstrates that the patient population and LAS have remained similar before and after 2017. In some respects, recipients of CLLTs after 2017 had better-preserved lung function as measured by FEV1. Suppose CLLT candidates represent a higher-risk population who are sicker and less likely to survive without transplantation compared to isolated lung transplant candidates. In that case, the LAS may have historically and currently been poor at capturing this degree of illness and risk level for this rare population. Combined with our finding that longer waitlist time is associated with an increased risk of post-CLLT mortality, the implementation of the new composite allocation system (CAS) for lung transplantation should be evaluated to address these issues.

Despite such limitations, dual-organ allocation in CLLT continues to be driven by the LAS instead of the MELD-XI scores [20, 21]. Similar to prior reports, the median CLLT recipient MELD-XI score at transplant was lower than most isolated liver transplant recipients [6, 12]. According to the most recent OPTN liver report, only 14.2% of all liver transplant recipients had a MELD-XI score of 14 or lower [22]. Prior studies have demonstrated that using LAS is beneficial in predicting survival in CLLT [12]. These results indicate that MELD scores may not accurately reflect severity of disease in multiorgan transplant candidates. With liver disease often occurring secondary to existing lung disease in CLLT patients, the implications of our data regarding MELD-XI scores are limited, because we do not know the predictive performance of MELD-XI in patients without known primary liver disease [3]. Beyond that, the discrepancy between the severity of lung disease versus liver disease in CLLT candidates highlights the persistent clinical challenges in equitable multiorgan allocation in the modern era. Reese et al. highlight two key principles relevant to transplant policy: equal access to all candidates and the justification of any disparities only if they benefit the least advantaged—a group for which no clear metric currently exists [23]. Organ allocation reform should aim to reduce disparities in waitlist survival without prioritizing multiorgan transplant candidates over single-organ candidates or enabling futile transplants.

This is particularly complex given the variability in allocation practices across organ combinations. In CLLT, candidates with high LAS but considerably lower MELD scores may receive priority over liver-only candidates with higher MELD scores, potentially increasing waitlist mortality for the latter. Considering the higher incidence of chronic lung allograft rejection relative to liver grafts, this raises questions about the prudent use of limited resources. While Goldberg et al. found no increase in waitlist mortality or drop-off for bypassed liver-alone candidates under multiorgan allocation policies [24], their study did not assess long-term outcomes, such as quality-adjusted life-years. As multiorgan transplant demand grows, there is a pressing need to develop robust, ethically sound allocation policies, along with ongoing improvements in patient selection and surgical technique.

An additional consideration in CLLT is the potential immunologic protection conferred by the liver allograft on the co-transplanted organ [21, 25, 26]. Pediatric and adult CLLT case series have reported lower rates of acute rejection and bronchiolitis obliterans syndrome (BOS), with some patients maintained on lower tacrolimus levels without rejection [25, 27]. Proposed mechanisms include liver-induced regulatory T-cell activation via hepatic myeloid cells, neutralization of lymphocytotoxic antibodies, and antigen load-induced immune tolerance [28–30]. Mortality is more often attributed to sepsis than rejection, and most rejection episodes are steroid responsive [9, 11, 18, 31]. While target levels of immunosuppression may be lowered in CLLT compared to isolated lung or liver transplants, further studies are needed to define optimal immunosuppressive strategies in CLLT.

Despite the correlation between higher LAS and greater survival benefits in CLLT recipients with cystic fibrosis [32], our study demonstrates that this may not be the case in recipients with non-cystic fibrosis indications for CLLT. Notably, patients diagnosed with IPF had the highest risk, experiencing a 4.74 times higher likelihood for mortality compared to those diagnosed with cystic fibrosis. This risk is likely mediated in part by the high risk of BOS and subsequent mortality in this population [33, 34]. BOS is the number one cause of post-transplant mortality after the first year in lung transplant recipients and is responsible for roughly 30% of yearly deaths afterward [35, 36]. Median survival following BOS diagnosis in bilateral lung transplant recipients is 30 months [37]. This same figure may be as low as 8 months in single lung transplant recipients diagnosed with IPF, suggesting that IPF patients have an even greater risk for mortality from BOS-related chronic lung allograft dysfunction [33]. Other factors involved in higher post-transplant mortality for IPF patients may include greater age at transplant and more significant age-related comorbidities [34, 36]. IPF is rarely diagnosed before age 50, with a mean age at diagnosis of around 65–70 years [38].

Although clinicians should be aware that IPF patients have a higher probability of post-transplant mortality, we do not believe this risk necessarily eliminates the viability of CLLT for IPF patients. Given this population’s unpredictable course of disease, high risk for developing lung cancer, and elderly age at diagnosis, IPF patients who may benefit from CLLT should be evaluated and listed for transplant early to maximize the survival benefit from surgery [34, 36]. Risk stratification aimed at optimizing candidate selection and post-transplant survival is likewise crucial. Major contraindications include recent tumor history, extreme obesity, unmanaged coronary artery disease, history of medical noncompliance, and insufficient social support [36]. CLLT recipients also have high levels of chronic colonization, especially among CF patients. Yi et al. recommended three months of continued antibiotic therapy post-CLLT [11], whereas Grannas et al. support a prophylactic antibiotic regimen, including inhalational and topical amphotericin B, ceftazidime/tobramycin/flucloxacillin for a minimum of 10 days, followed by life-long 800/160-mg trimethoprim/sulfamethoxazole and oral itraconazole [9]. Cytomegalovirus (CMV) also poses a threat to CLLT recipients. However, there is no universal antiviral prophylaxis regimen for “at-risk” CLLT recipients, aside from routine testing for CMV cytopathic changes or CMV antigen [1].

### Limitations

Our study has several limitations. First, this is a retrospective analysis and, thus, subject to inherent selection bias. While patients with missing or incomplete data entry were excluded from our study, patient selection for CLLTs may vary based on institutional policies and individual clinicians’ decision-making. These factors introduce potential confounders that we were unable to consider. Second, the increase in CLLTs performed in 2020 coincided with the COVID-19 pandemic, which may have influenced patient selection, donor availability, and perioperative management in ways not fully accounted for in the analysis. Lastly, our study is limited by the rarity of the procedure. While this report represents one of the largest cohorts of CLLT recipients in the modern era—providing sufficient power to perform large multivariate analyses—the lack of statistical significance in certain variables may be due to the insufficient power to identify a potential association. Given the rarity and highly selected nature of the CLLT population, propensity score matching was not feasible due to the limited sample size and lack of suitable control subjects with comparable clinical profiles. Instead, we used multivariable Cox regression modeling to adjust for 10 major confounding variables with biological plausibility.

## Conclusion

In the last decade, CLLTs have been increasingly performed with comparable short- and long-term survival to isolated lung transplantation. Since the 2017 geographic allocation change to LAS, recipient and lung allograft survival up to 3 years as well as acute rejection rates have largely remained similar after CLLT. This is despite an increase in donor organ ischemic times and travel distance to recipient transplant centers. Higher LAS, but not MELD-XI scores continue to represent the primary driver for increased risk of mortality, particularly among patients with non-cystic fibrosis indication for transplant, including IPF, obstructive lung disease, A1 AD, pulmonary hypertension, sarcoidosis, and COVID-19-related ARDS. Thus, while our study demonstrates that carefully selected candidates may benefit from CLLTs in the long run, the national guidelines for this procedure should be continually evaluated as the new CAS system replaces the LAS.

## Supplementary Information

Below is the link to the electronic supplementary material.

Supplementary file1 (PDF 120 KB)

## Data Availability

The data that support the findings of this study were obtained from the United Network for Organ Sharing (UNOS) Standard Analysis and Research (STAR) database. Instructions for accessing this database can be found at https://optn.transplant.hrsa.gov/data/view-data-reports/request-data/data-request-instructions/.
