# Supplementary material for: National Trends and Outcomes of Combined Lung–Liver Transplantation: An Analysis of the UNOS Registry
Source: Lung. 2025 Apr 25;203(1):57. doi: 10.1007/s00408-025-00811-9 (PMC12031901; doi:10.1007/s00408-025-00811-9)
Supplement: Supplementary file 1 — Supplementary file1 (PDF 120 KB) [file 408_2025_811_MOESM1_ESM.pdf]

Title: National Trends and Outcomes of Combined Lung-Liver Transplantation: An Analysis of the UNOS Registry.

Journal Name: LUNG

Authors: Brian J. Bao, BS[1], Ye In Christopher Kwon, BA[1], Emily G. Dunbar, MD[1], Zachary Rollins, MD[1], Jay Patel, MD[1], Matthew Ambrosio, MS[2], David A. Bruno, MD[3], Vipul Patel, MD[4], Walker A. Julliard, MD[1], Vigneshwar Kasirajan, MD[1], Zubair A. Hashmi, MD[1]

Affiliations:

1. Division of Cardiothoracic Surgery, Department of Surgery, Pauley Heart Center, Virginia Commonwealth University School of Medicine, Richmond, VA, USA
2. Department of Biostatistics, Virginia Commonwealth University School of Population Health, Richmond, VA, USA
3. Division of Abdominal Transplant Surgery, Department of Surgery, Hume-Lee Transplant Center, Virginia Commonwealth University School of Medicine, Richmond, VA, USA
4. Division of Pulmonary Disease and Critical Care Medicine, Department of Internal Medicine, Virginia Commonwealth University School of Medicine, Richmond, VA, USA

Corresponding Author:

Brian J Bao, BS

bjb2kva@virginia.edu

The below supplementary material includes two Kaplan-Meier survival curves comparing all adults undergoing combined lung-liver and isolated lung transplantation in the United States in era 1 (Fig. S1) and in era 2 (Fig. S2)

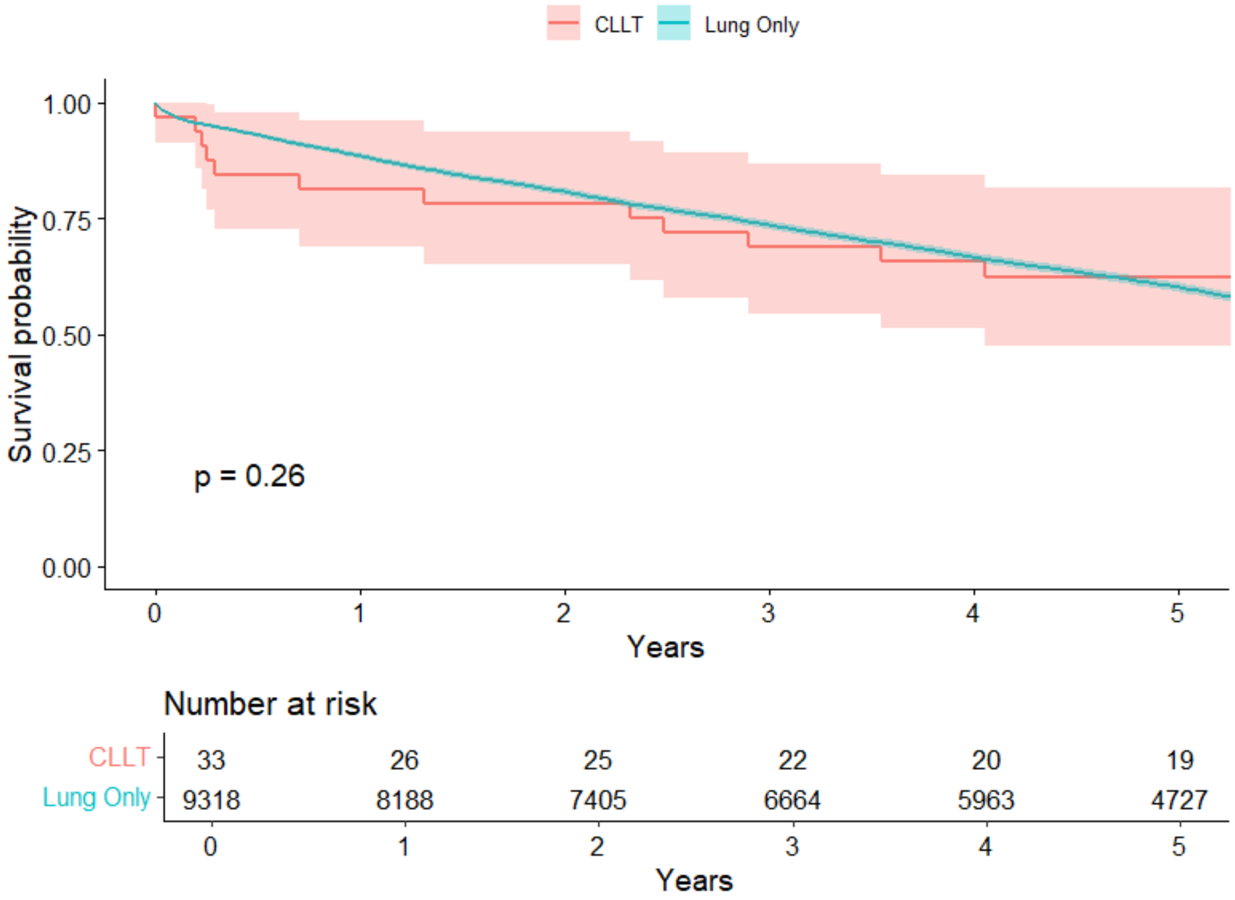

Fig. S1 Kaplan-Meier survival curve comparing all adults undergoing combined lung-liver and isolated lung transplantation in the United States within era 1. Era 1 is from January 1, 2014 to November 24, 2017. Shaded regions represent the 95% confidence interval

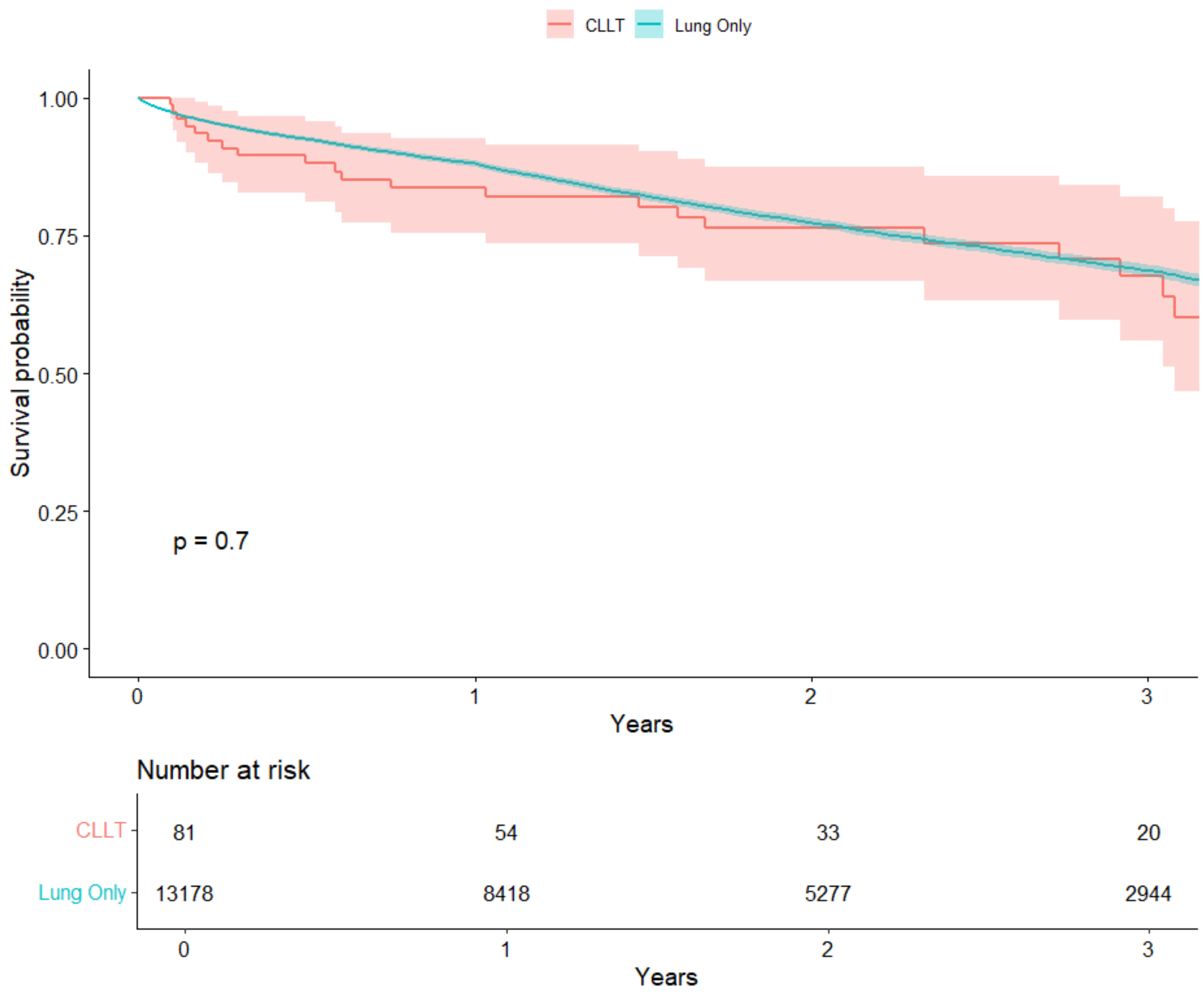

Fig. S2 Kaplan-Meier survival curve comparing all adults undergoing combined lung-liver and isolated lung transplantation in the United States within era 2. Era 2 is from November 25, 2017 to June 30, 2023. Shaded regions represent the 95% confidence interval
